# Identification of two terpenoids that accumulate in Chinese water chestnut in response to fresh‐cut processing

**DOI:** 10.1002/fsn3.3475

**Published:** 2023-06-12

**Authors:** Hui Nie, Yanghe Luo, Shuangquan Huang, Yuwei Mo, Zhenli Huang, Yuemei Liao, Lirui Jiang, Wen Cai, Mubo Song

**Affiliations:** ^1^ Research Institute of Food Science and Engineering Technology Hezhou University Hezhou China; ^2^ School of Food Science and Technology Dalian Polytechnic University Dalian China

**Keywords:** Chinese water chestnut, purification, secondary metabolism, structural identification, terpenoid biosynthesis

## Abstract

As a form of vegetable in China, freshly cut corms of Chinese water chestnuts (*Eleocharis dulcis*) are well received by consumers. Few studies have investigated the metabolites present in fresh‐cut *E. dulcis*, particularly during the storage stage. Two compounds, triterpenoids and apocarotenoids, were identified in fresh‐cut *E. dulcis* during the late storage period using thin‐layer chromatography (TLC), high‐performance liquid chromatography (HPLC), and nuclear magnetic resonance (NMR) spectroscopy. The content of these two compounds gradually increased in the surface tissue of fresh‐cut *E. dulcis* during storage. Moreover, the transcript levels of 10 genes involved in terpenoid backbone biosynthesis and five genes involved in carotenoid precursor biosynthesis were evaluated via quantitative real‐time PCR (qRT‐PCR). Expression of the rate‐limiting enzyme‐coding genes *CwDXS* and *CwHMGS* was significantly induced by wounding. CwMYC and CwbHLH18, which belong to bHLH transcription factors (TFs) IIIe and VIa subgroup, were isolated from *E. dulcis* corm. Phylogenetic analysis showed that CwMYC and CwbHLH18 grouped with other terpenoid‐regulated bHLHs, and their transcript levels were strongly induced after fresh‐cut processing. These results suggested that the biosynthesis of terpenoids and apocarotenoids in fresh‐cut *E. dulcis* strongly depended on the transcriptional regulation of structural genes involved in the methylerythritol 4‐phosphate (MEP) and mevalonate (MVA) pathways. However, the complex secondary metabolism of fresh‐cut *E. dulcis* during late storage requires further investigation.

## Graphical abstract

6ß‐Hydroxystigmast‐4‐en‐3‐one and blumenol A were isolated and identified from CWC for the first time. Fresh‐cut processing induced the biosynthesis of these two compounds in CWC during the late stage of storage. Expression of structural genes involved in terpenoid backbone biosynthesis and carotenoid precursor biosynthesis of CWC was induced by wounding.

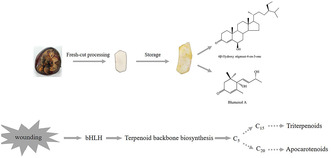

## 1 INTRODUCTION

Most fresh‐cut fruit and vegetable products rapidly lose their freshness and typical flavor during storage (Barrett et al., 2010). In addition, wounding induces metabolism, and many secondary metabolites that are not detectable in intact tissues accumulate after fresh‐cut processing (Wang et al., 2015). *Eleocharis dulcis* (Burm.f.) Trin. ex Hensch. commonly known as Chinese water chestnut, is an aquatic plant form the sedge family (Cyperaceae). Corms can be eaten fresh or used as vegetables, and are widely used in folk medicine (Luo et al., 2014). Fresh‐cut *E. dulcis* is becoming a popular ready‐to‐eat or ready‐to‐use product because of its high nutritional value and unique taste. Its use as a convenience product in China is much more common than that of intact corm in retail markets (Peng & Jiang, 2006). However, fresh‐cut processing reduces product shelf life and causes stress due to the mechanical wounding of tissues resulting in the breakdown of texture, discoloration of cut surfaces and changes differences in flavor and nutritional quality (Mashabela et al., 2019). Due to damage caused by processing, fresh‐cut *E. dulcis* is highly perishable.

Changes in the components of fresh‐cut products are caused by changes in enzymatic activities and physiological metabolism, which are accompanied by visual spoiling (Yousuf et al., 2018). A previous study demonstrated that flavonoid biosynthesis was induced by wounding, and after a few days of storage, various flavonoids accumulated on the surface tissue of fresh‐cut *E. dulcis* (Li et al., 2016; Song, Shuai, et al., 2019). The major chemicals involved in the yellowing of fresh‐cut *E. dulcis* have been identified as the two flavonoids eriodictyol and naringenin (Pan et al., 2015; Song, Wu, et al., 2019). Similar to flavonoids, the content of other intermediate products in different branches of the phenylpropanoid pathway increases dramatically during storage (Li et al., 2016). Phenolic compounds have been intensively studied as major secondary metabolites in fresh‐cut *E. dulcis* during the early storage period (Pan et al., 2015). However, the metabolic processes within fresh‐cut *E. dulcis* can be more complex than those within traditional fresh‐cut vegetables and are still not fully understood, especially during the late storage period.

Therefore, the status of metabolites newly synthesized via different pathways after fresh‐cut processing must be evaluated. The purpose of this study was to investigate the chemicals in fresh‐cut *E. dulcis* during the late stage of storage and discuss wound‐induced metabolic variations.

## 2 MATERIALS AND METHODS

### 2.1 Plant material

Corms of *Eleocharis dulcis* (Burm.f.) Trin. ex Hensch. (Cyperaceae) were obtained from a farmers market in Hezhou city, Guangxi Province of China. Corms with uniform shape, size, and no mechanical injury or signs of disease were selected. The corms were sterilized with sodium hypochlorite solution (0.01%, v/v), washed, peeled, and cut into slices (4–5‐mm‐thick slices). The slices were put into plastic pallets wrapped with polyethylene film (0.02 mm thick), and then stored at 15 ± 1°C. Different tissues, including tubular stems, stem bases, corm peels, corm flesh and roots, were harvested from the *E. dulcis* at the swelling stage. All the samples were frozen in liquid nitrogen and stored at −80°C until used.

### 2.2 Extraction and isolation of compounds

*Eleocharis dulcis* tissue (10 kg) that had been stored for 6 days at 15 ± 1°C was extracted three times with 75% ethanol (25 L × 3), each time for 24 h, at room temperature. The resulting ethanol extract was evaporated in vacuo, yielding a residual mass (47 g). The residual mass was separated into EtOAc (3 × 6 L), *n‐*BuOH (3 × 6 L), and H_2_O layers using liquid–liquid partitioning. The EtOAc extract (24 g) was divided into six fractions (A–F) by loading it onto a silica gel CC (8 i.d. × 30 cm) and eluting it through a gradient of petroleum ether/EtOAc (100:0, 95:5, 90:10, 80:20: 70:30, 0:100, v/v). Fraction A (6 g) was loaded onto a silica gel CC (3 i.d. × 10 cm), eluted through a gradient of petroleum ether/EtOAc (100:0, 90:10, 85:15, 70:30, 0:100, v/v) and purified using Sephadex LH‐20 CC (2 i.d. × 100 cm, CC, Amersham Pharmacia Biotech AB), yielding compound **1** (5 mg). Fraction C (5.4 g) was separated on another silica gel column (3 i.d. × 10 cm CC, Amersham Pharmacia Biotech AB) through a gradient of petroleum ether/EtOAc (100:0, 90:10, 80:20, 70:30, 0:100, v/v), yielding compound **2** (6.5 mg). The protocol for the isolation and purification of these two compounds is shown in Figure S1.

### 2.3 Isolated terpenoids

#### 2.3.1 6β‐Hydroxystigmast‐4‐en‐3‐one

We isolated compound 1 (5 mg), which was colorless and acicular. The ^1^H‐NMR (500 MHz, DMSO‐d6) were as follows: 5.65 (1H, *s*, H‐4), 4.14 (1H, *s*, H‐6), 2.41 (2H, *m*, H‐7), 1.98 (1H, *m*, H‐25), 1.83 (3H, *s*, H‐19), 1.36 (1H, *s*, H‐20), 1.28 (3H, *s*, H‐21), 1.21 (2H, *m*, H‐7), 1.19 (2H, *m*, H‐28), 0.90 (3H, *s*, H‐29), 0.82 (3H, *s*, H‐27), 0.80 (3H, *s*, H‐26), and 0.69 (3H, *s*, H‐18). The ^13^C‐NMR (126 MHz, DMSO‐*d*_6_) results were as follows: 199.2 (C‐3), 169.1 (C‐5), 125.1 (C‐4), 71.1 (C‐6), 55.5 (C‐14), 55.3 (C‐17), 53.1 (C‐9), 45.1 (C‐24), 42.0 (C‐13), 38.8 (C‐12), 37.5 (C‐7), 36.6 (C‐10), 35.5 (C‐1), 33.9 (C‐20), 33.3 (C‐2), 29.4 (C‐22), 28.7 (C‐8), 27.8 (C‐25), 25.5 (C‐16), 23.8 (C‐23), 22.6 (C‐15), 20.5 (C‐28), 19.7 (C‐11), 18.9 (C‐26), 18.8 (C‐19), 18.6 (C‐27), 11.8 (C‐21), and 11.7 (C‐18). The 1H‐NMR and 13C‐NMR spectra of the compounds are shown in Figures S2 and S3.

#### 2.3.2 Blumenol A

Compound 2 (6.5 mg) was isolated as white needles. The results ^1^H‐NMR (500 MHz, DMSO‐*d*_6_) results were as follows: 5.78 (1H, *s*, H‐4), 5.68 (1H, *m*, H‐8), 5.60 (1H, *d*, H‐7), 4.99 (1H, *s*, ‐OH), 4.72 (1H, *s*, ‐OH), 4.18 (1H, *s*, H‐9), 2.36 (1H, *d*, H‐2a), 2.06 (1H, *d*, H‐2b), 1.81 (3H, *s*, H‐13), 1.11 (3H, *s*, H‐10), 0.94 (3H, *s*, H‐12) and 0.91 (1H, *s*, H‐11). The ^13^C‐NMR (126 MHz, DMSO‐*d*_6_) results were as follows: 197.2 (C‐3), 164.0 (C‐5), 135.9 (C‐7), 127.9 (C‐8), 125.5 (C‐4), 77.8 (C‐6), 66.1 (C‐9), 49.4 (C‐2), 41.0 (C‐1), 24.2 (C‐10), 24.1 (C‐12), 23.9 (C‐11), 23.1 (C‐12), and 18.9 (C‐13). The ^1^H‐NMR and ^13^C‐NMR spectra of the compounds are shown in Figures S4 and S5.

### 2.4 Quantification of the compounds

The contents of these two compounds were then measured by high‐performance liquid chromatography (HPLC). The *E. dulcis* tissues sampled at 0, 3, and 6 days (15 g) were extracted at room temperature with ethyl acetate (1:2, m/v) for 24 h. The mixture was then dried using rotary evaporation (Eyela, Japan) and redissolved in 5 ml of methyl alcohol. The supernatant was passed through a 0.45 μm‐Millipore filter, and then the sample (10 μL) was injected into Shimadzu Prominence‐i LC2030C HPLC (Japan) system equipped with a variable‐wavelength PDA detector. Compound 1 was separated on an Agilent Extend C18 column (4.6 × 250 mm, 5 μm) at 27°C. A gradient HPLC system was eluted with eluent A (water) and eluent C (methyl alcohol). The elution profile was as follows: 0–10 min A followed by 100% C. The flow rate of the mobile phase was 1.0 ml min^−1^, and tr = 7.84 min. Compound 2 was separated on an Agilent Extend C18 column (4.6 × 250 mm, 5 μm) at 20°C. The elution profile was as follows: 0–8 min A followed by 20%–100% C. The flow rate of the mobile phase was 0.8 ml min^−1^, and tr = 6.92 min. The metabolites within the samples were identified and quantified by comparison with peak retention time and peak area from standards. The content of two compounds was expressed as μg g^−1^ fresh weight (FW).

### 2.5 Expression analyses of genes related to terpenoid and carotenoid biosynthesis

The full‐length cDNA of 10 structural genes involved in terpenoid biosynthesis and five genes involved in carotenoid biosynthesis was isolated from *E. dulcis* corms. Specifically, total RNA (1000 ng) from *E. dulcis* corms was reverse transcribed using a PrimeScript RT Reagent Kit (TaKaRa, Japan). The partial coding DNA sequences (CDSs) of these genes were derived from transcriptome data of *E. dulcis* (Song, Shuai, et al., 2019), and full‐length cDNA clones were ultimately obtained using a SMARTer rapid amplification of cDNA ends (RACE) 5′/3′ Kit (TaKaRa, Japan). Sequence data from this article can be found in the GenBank data library under accession numbers MN597406 to MN597415 (for *CwDXS*, *CwDXR*, *CwCMK*, *CwMDS*, *CwHDS*, *CwHDR CwHMGS*, *CwHMGR*, *CwPMK*, and *CwMVD*, respectively), MN597416 to MN597420 (for *CwPSY1*, *CwPSY2*, *CwPSY3*, *CwPDS*, and *CwZDS*, respectively) and MT468375 and MT468376 (for *CwMYC* and *CwBHLH18*). Quantitative real‐time PCR (qRT‐PCR) assay was carried out according to Song, Wu, et al. (2019). In brief, the qRT‐PCR was set up with SYBR Green qPCR Master Mix (TaKaRa, Japan) and performed in a CFX Connect Real‐Time Detection System (Bio‐Rad, USA). Data were normalized to reference gene18S rRNA of *E. dulcis* (MG742686). Primers used for qRT‐PCR assay are listed in Table S1.

## 3 RESULTS AND DISCUSSION

### 3.1 Quantification of 6β‐Hydroxystigmast‐4‐en‐3‐one and blumenol A during storage

**FIGURE 1:**
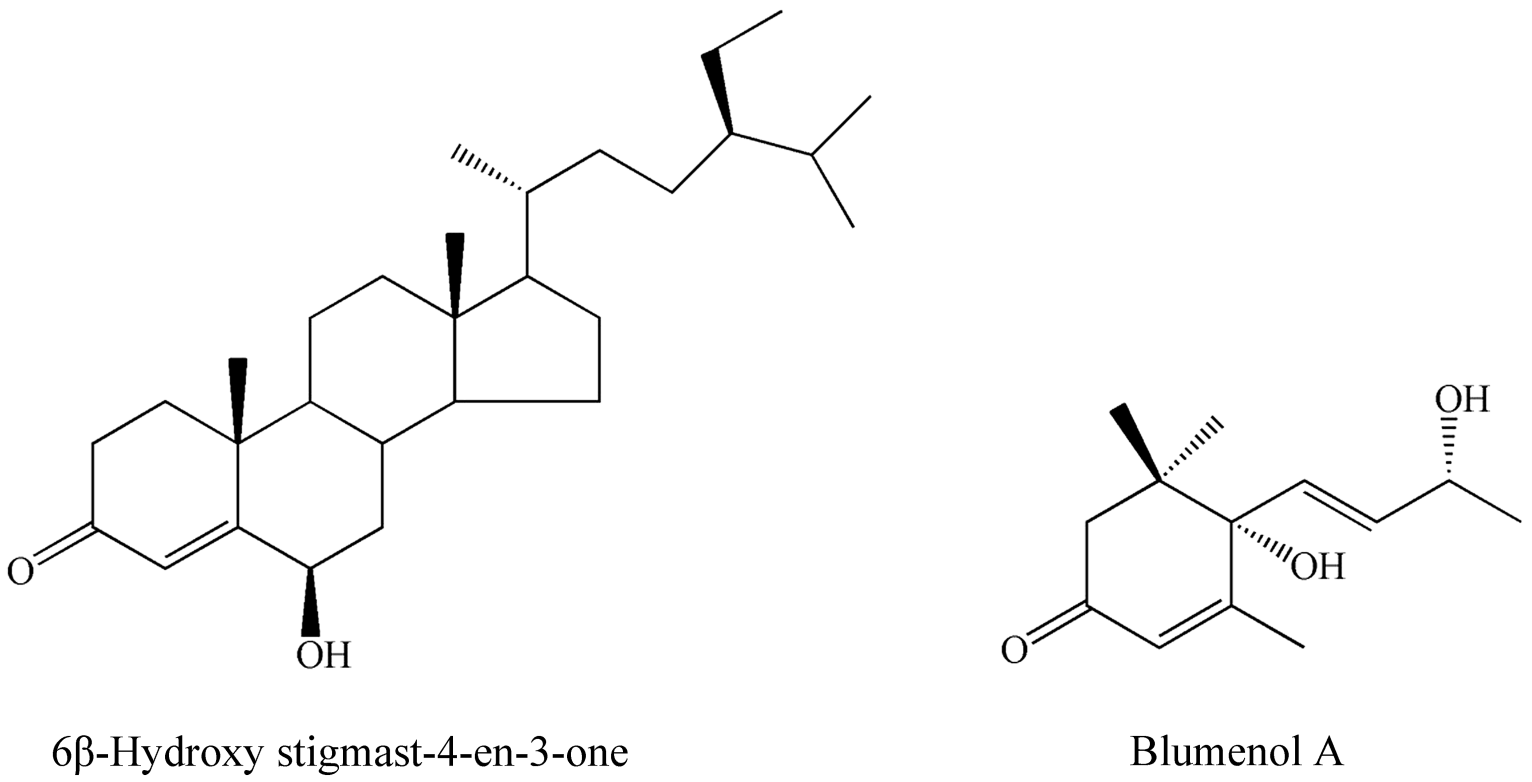
Chemical structures of compounds 1 and 2 isolated from fresh‐cut *Eleocharis dulcis*.

Preliminary studies showed that these two terpenoids accumulate during storage. Isolation and structural analysis using nuclear magnetic resonance (NMR) identified these as the triterpene 6β‐Hydroxystigmast‐4‐en‐3‐one 1 (Figure 1) by comparison with the spectral data in the literature (Georges et al., 2006) and the carotenoid derivative (+/−)‐6‐hydroxy‐3‐oxo‐alpha‐ionol, commonly called blumenol A, 2 (Figure 1) by comparison with the spectral data in the literature (Niu et al., 2003).These compounds have been isolated from several plant species (Lee et al., 2007; Llanos et al., 2010; Qi et al., 2014) but not from Cyperaceae.

**FIGURE 2:**
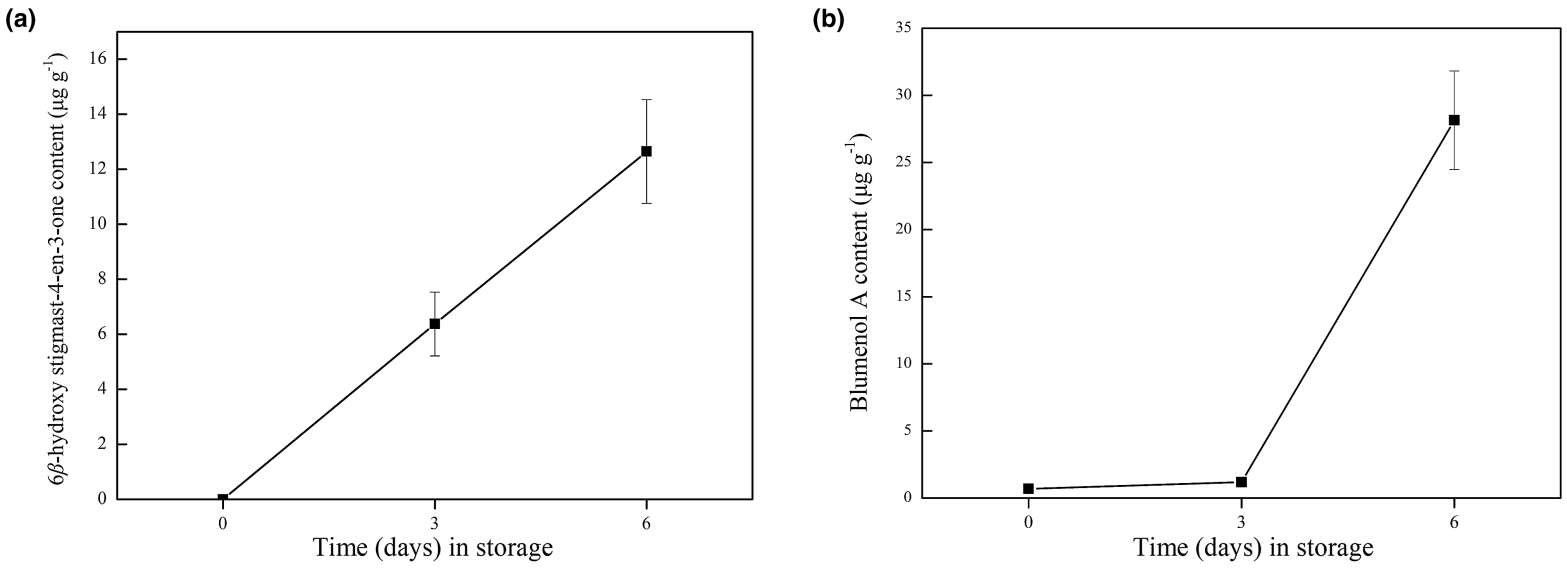
Time course of the 6β‐Hydroxystigmast‐4‐en‐3‐one and blumenol A accumulation in fresh‐cut *Eleocharis dulcis* during storage.

The content of 6β‐Hydroxystigmast‐4‐en‐3‐one and blumenol A in fresh‐cut *E. dulcis* was determined using HPLC during storage. As shown in Figure 2a, 6β‐Hydroxystigmast‐4‐en‐3‐one levels in fresh‐cut *E. dulcis* were undetectable on day 0. After prolonged storage, the content of 6β‐Hydroxystigmast‐4‐en‐3‐one increased. Blumenol A content was low at the beginning of storage but accumulated to high levels after 6 days of storage (Figure 2b). Different plant species and tissues synthesize hundreds of terpenoids (Pichersky & Raguso, 2018). In many plant species, the accumulation of terpenoid compounds increases in response to various biotic and abiotic stresses, such as insect attacks and mechanical wounding (Martin et al., 2002; Pateraki & Kanellis, 2010). Our results suggest that fresh‐cut processing induces the biosynthesis and accumulation of both 6β‐Hydroxystigmast‐4‐en‐3‐one and blumenol A.

### 3.2 Expression analysis of genes related to the terpene pathway during fresh‐cut *E. dulcis* storage

**FIGURE 3:**
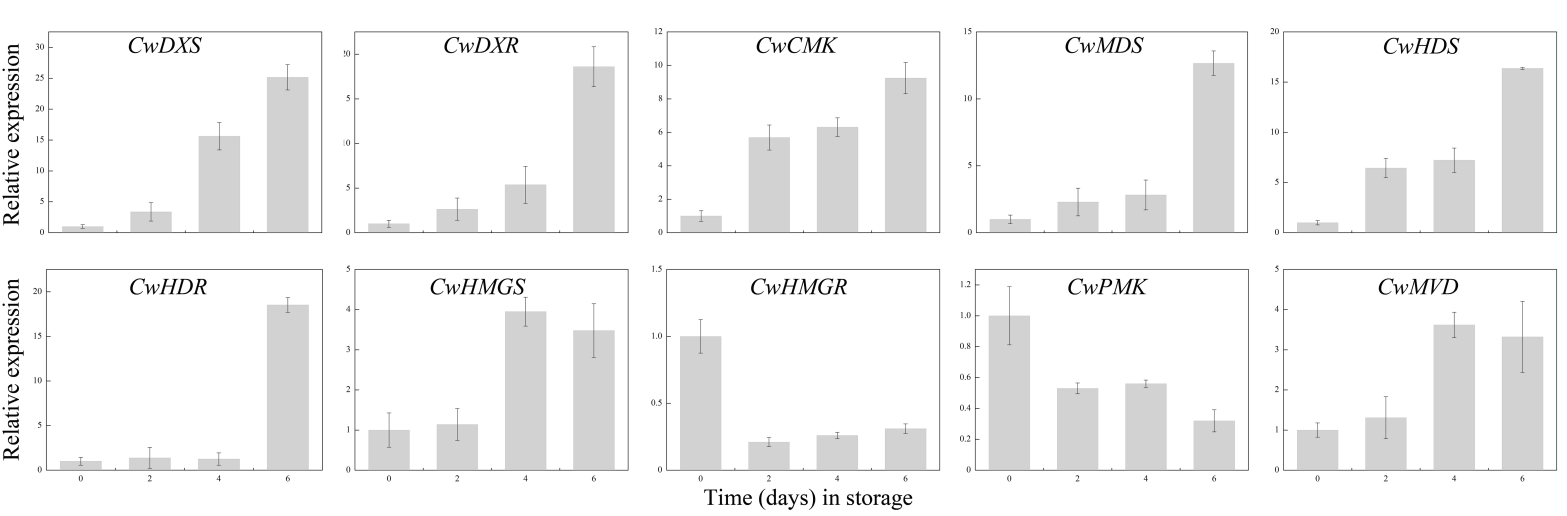
Expression of genes that encode key enzymes involved in terpenoid backbone biosynthesis in *Eleocharis dulcis* during storage. Total RNA was extracted from fresh‐cut *E. dulcis* 0, 2, 4, and 6 days after peeling. The expression data were normalized against the expression of 18S rRNA, and gene expression levels were set to 1 in the day 0 samples. Each column represents the mean ± SE of three biological replicates.

Terpenoids, with many relevant pharmacological properties, are the most important group of secondary metabolites (Montesano et al., 2018). The mevalonate (MVA) and methylerythritol 4‐phosphate (MEP) pathways are involved in the biosynthesis of the five‐carbon terpenoid backbone (Nagegowda, 2010). 3‐Hydroxy‐3‐methyl‐glutaryl‐CoA reductase (HMGR, EC 1.1.1.88) and 1‐deoxy‐D‐xylulose‐5‐phosphate synthase (DXS, EC 2.2.1.7) are rate‐limiting enzymes in the MVA and MEP pathways, respectively (Chappell et al., 1995; Lois et al., 2000). Biosynthesis of the five‐carbon terpenoid backbone compensates for the precursors needed for downstream terpenoid and apocarotenoid biosynthesis. Terpenoids are involved in the plant response to a wide range of biotic and abiotic stresses, including mechanical wounding (Balusamy et al., 2015). The mechanical injury incurred while processing fresh‐cut fruits and vegetables produces a signal that induces the accumulation of various metabolites (Song, Wu, et al., 2019; Yousuf et al., 2018). The biosynthesis of secondary metabolites in plants is developmentally controlled, and this regulation primarily depends on the transcriptional control of enzyme‐coding genes (Chezem & Clay, 2016). For the first time, 10 full‐length structural genes involved in terpenoid synthesis in *E. dulcis* were isolated. The expression patterns of the cloned genes were investigated using qRT‐PCR in fresh‐cut *E. dulcis* tissues during storage. The results showed that the expression levels of genes involved in the MEP pathway, including *CwDXS*, *CwDXR*, *CwCMK*, *CwMDS*, *CwHDS*, and *CwHDR*, were dramatically upregulated after the corms were peeled and cut and increased gradually with prolonged storage time (Figure 3). However, the genes involved in the MVA pathway exhibited diverse expression patterns during storage. Under our experimental conditions, the expression of *CwHMGS* and *CwMVD* was upregulated after 4 days of storage, whereas the transcript levels of *CwHMGR* and *CwPMK* decreased gradually with extended storage time (Figure 3). In plants, the MVA pathway provides precursors for sesquiterpenes and sterols, whereas the MEP pathway produces monoterpenes, diterpenes, and carotenoids (Bartram et al., 2006). The results of the present study suggested that mechanical wounding enhanced the biosynthesis of terpenoids in the corms of *E. dulcis* and that the expression of the rate‐limiting enzyme‐coding genes *CwDXS* and *CwHMGS* was induced by wounding.

### 3.3 Expression analysis of genes related to apocarotenoid biosynthesis during storage of fresh‐cut *E. dulcis*

**FIGURE 4:**
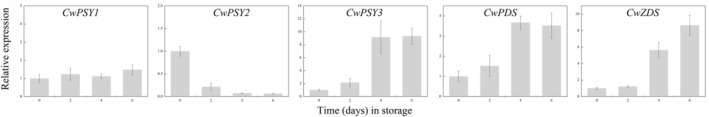
Expression of genes that encode key enzymes involved in carotenoid biosynthesis in *Eleocharis dulcis* during storage. Total RNA was extracted from fresh‐cut *E. dulcis* 0, 2, 4, and 6 days after peeling. The expression data were normalized against the expression of 18S rRNA, and gene expression levels were set to 1 in the day 0 samples. Each column represents the mean ± SE of three biological replicates.

In plants, blumenol A is an apocarotenoid derivative derived from the cleavage of C_40_ carotenoid precursors. This process is accompanied by the production of the C14 compound mycorradicin (Hou et al., 2016). Blumenol accumulates at high levels in the roots of many plant species, and its antifungal and phytotoxic properties have been studied (Fester et al., 2002; Kato‐Noguchi et al., 2012; Vierheilig et al., 2000; Walla et al., 2000). It is reasonable to assume that apocarotenoid formation requires constitutive carotenoid precursor pools (Walter et al., 2015). However, little is known about the origin of carotenoid precursors in non‐photosynthetic plant tissues. Carotenoid levels are very low in the corm of *E. dulcis*, and there is little information on corm‐specific carotenoid biosynthesis. However, several studies have shown that environmental factors may influence the availability of isoprenoid precursors and that carotenoid biosynthesis is associated with stress conditions (Cazzonelli & Pogson, 2010; Perrin et al., 2017). Therefore, to determine whether the expression of genes involved in the biosynthesis of carotenoid precursors was induced by wounding, we monitored the changes in the expression of genes involved in carotenogenesis in fresh‐cut *E. dulcis*, such as *CwPSY*, *CwPDS*, and *CwZDS*. Wounding caused similar expression patterns in *CwPDS* and *CwZDS*. The transcription of these two genes was slightly induced after peeling, and increased continuously with prolonged storage (Figure 4). PSY, encoded by a member of a small gene family, catalyzes the first committed step in carotenoid biosynthesis (Gallagher et al., 2004). Three *PSY* genes were isolated and clustered into two groups (Figure S6). Notably, the expression patterns of the three *PSY* genes in fresh‐cut *E. dulcis* varied during storage. The transcription of *CwPSY3* significantly increased after peeling, whereas the expression of *CwPSY2* was downregulated during storage. The transcription of *CwPSY1* did not respond to these conditions. It is well known that different members of the *PSY* family exhibit different tissue‐specific expression patterns in many plant species (Li et al., 2008; Peng et al., 2013). The accumulation of apocarotenoids and their derivatives requires sufficient amounts of carotenoid precursors. However, most leucoplasts in non‐photosynthetic and white plant tissues, such as roots, tubers, and corms, have little capacity for the biosynthesis and storage of carotenoids (Li & Yuan, 2013; Parry & Horgan, 1992). Similarly, all members of the *PSY* family were highly expressed in the photosynthetic tissues of *E. dulcis* (Figure S7). In this study, the expression of carotenoid synthesis genes played a role in controlling carotenoids flux in the corms of *E. dulcis* in response to wounding. Members of the *PSY* gene family vary in tissue specificity concerning their expression and in response to abiotic stress. We found that *CwPSY3* regulates wound‐induced carotenogenesis in corms and may provide sufficient carotenoid precursors for apocarotenoid production.

### 3.4 Expression analysis of CwbHLHs grouped in subgroup IIIe and IVa during storage of fresh‐cut *E. dulcis*

**FIGURE 5:**
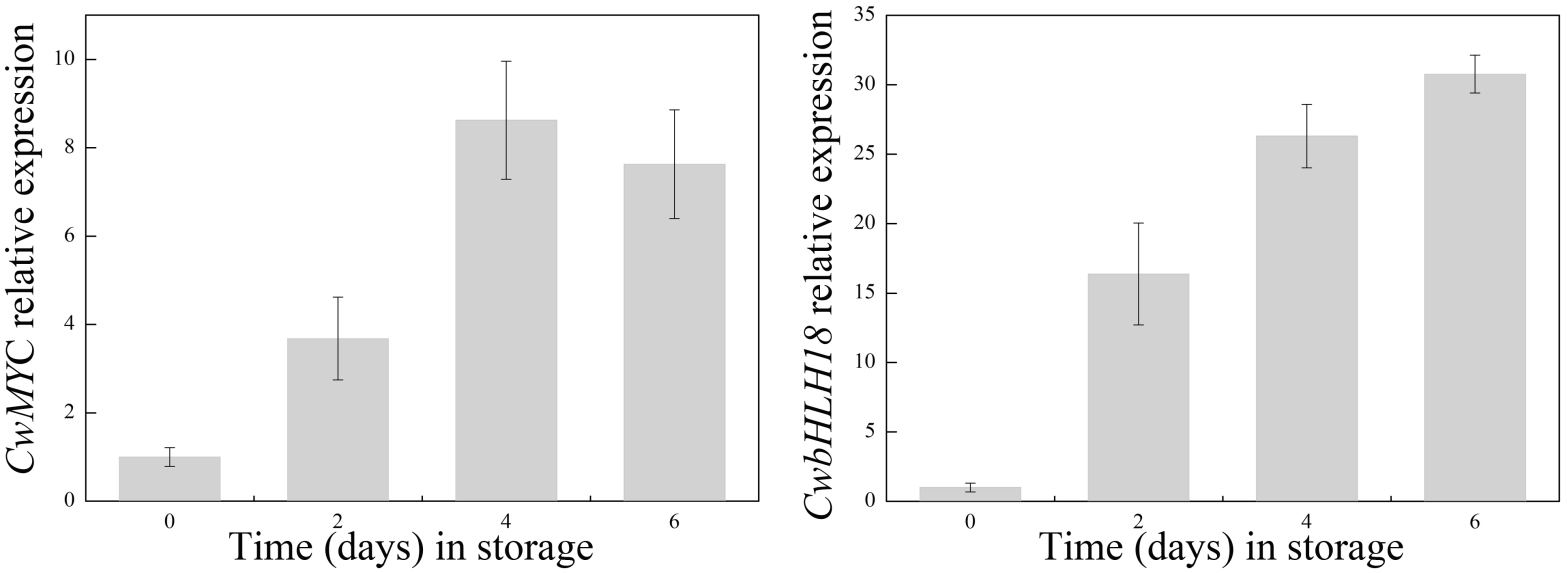
Changes in transcript levels of *CwMYC* and *bHLH18* in *Eleocharis dulcis* during storage. Total RNA was extracted from fresh‐cut *E. dulcis* 0, 2, 4, and 6 days after peeling. The expression data were normalized against the expression of 18S rRNA, and gene expression levels were set to 1 in the day 0 samples. Each column represents the mean ± SE of three biological replicates.

Although the structural genes involved in terpenoid biosynthesis have been extensively studied, the regulatory mechanisms of terpenoid biosynthesis remain largely unknown (Chuang et al., 2018). It has been shown that subgroups IIIe and VIa of bHLH transcription factors (TFs) are involved in terpenoid biosynthesis (Hong et al., 2012; Jan et al., 2016; Zhang et al., 2011). MYCs belonging subgroup to IIIe of bHLH TFs are central players in the primary JA signaling cascade. There is evidence that MYC TFs can directly modulate terpenoid biosynthesis by regulating genes in the MEP pathway. For instance, heterologous overexpression of *AtMYC2* significantly activates the expression of genes *DXS*, *DXR*, and *HDS* in the MEP pathway and the accumulation of downstream terpenoids in *Salvia sclarea* (Alfieri et al., 2018). In *Catharanthus roseus*, two Clade IVa bHLH TFs, namely CrBIS1 and CrBIS2, regulate terpenoid biosynthesis (Mertens et al., 2016). In the present study, two bHLH TFs (CwMYC2 and CwbHLH18) from *E. dulcis* corms, which were closely related to other regulators of terpenoid biosynthesis, clustered in subgroups IIIe and VIa, respectively (Figure S8). The qRT‐PCR results showed that the transcript levels of CwMYC2 and CwbHLH18 in fresh‐cut *E. dulcis* sharply increased during storage, consistent with the production of terpenoids and the expression patterns of structural genes in MVA and MEP pathway (Figure 5). The IIIe and VIa subgroups of bHLH TFs that regulate terpenoid biosynthesis are wound responsive (Jan et al., 2016). Our previous study demonstrated that genes involved in jasmonate biosynthesis were upregulated in fresh‐cut *E. dulcis* by wounding (Song, Shuai, et al., 2019). These results suggest that wound‐inducible bHLH TFs play an important role in activating terpenoid biosynthesis pathways in fresh‐cut *E. dulcis*.

## 4 CONCLUSION

In the present study, we isolated and identified two compounds, 6β‐Hydroxystigmast‐4‐en‐3‐one and blumenol A, from fresh‐cut *E. dulcis* during late storage. To the best of our knowledge, these two compounds have not previously been reported in any Cyperaceae species. Fresh‐cut *E. dulcis* is a traditional fresh food product; however, relatively long storage periods are required in food markets. The metabolism of the fresh‐cut corm of *E. dulcis* is more complex than that of other traditional fresh‐cut vegetables. This is the first report indicating that terpenoid biosynthesis was enhanced and that the transcript levels of related genes were upregulated in fresh‐cut *E. dulcis* in response to wounding under prolonged storage. Accumulation of specific terpenoids and apocarotenoids constantly alters the composition of fresh‐cut *E. dulcis*. These metabolites' biological functions and biosynthetic mechanisms have been gradually clarified; however, their effects on human health are less clear, and additional studies are needed.

## AUTHOR CONTRIBUTIONS

**Hui Nie:** Data curation (lead); formal analysis (lead); writing – review and editing (lead). **Yanghe Luo:** Funding acquisition (equal); investigation (equal); methodology (equal). **Shuangquan Huang:** Data curation (equal); formal analysis (equal); investigation (equal); methodology (equal). **Yuwei Mo:** Formal analysis (equal); funding acquisition (equal); investigation (equal). **zhenli Huang:** Data curation (equal); formal analysis (equal); funding acquisition (equal). **Lirui Jiang:** Investigation (equal); methodology (equal); writing – review and editing (equal). **Yuemei Liao:** Data curation (equal); formal analysis (equal); funding acquisition (equal); investigation (equal). **Wen Cai:** Funding acquisition (equal). **Mubo Song:** Conceptualization (lead); writing – original draft (lead).

## CONFLICT OF INTEREST STATEMENT

The authors declare that they have no conflict of interest.

## Supporting information


Figure S1

Figure S2

Figure S3

Figure S4

Figure S5

Figure S6

Figure S7

Figure S8

Table S1

## Data Availability

The datasets used and/or analyzed during this study are available from the corresponding author upon reasonable request.
