# Supplementary figures and images for: Identification of two terpenoids that accumulate in Chinese water chestnut in response to fresh‐cut processing
Source: Food Sci Nutr. 2023 Jun 12;11(9):5166–73. doi: 10.1002/fsn3.3475 (PMC10494652; doi:10.1002/fsn3.3475)

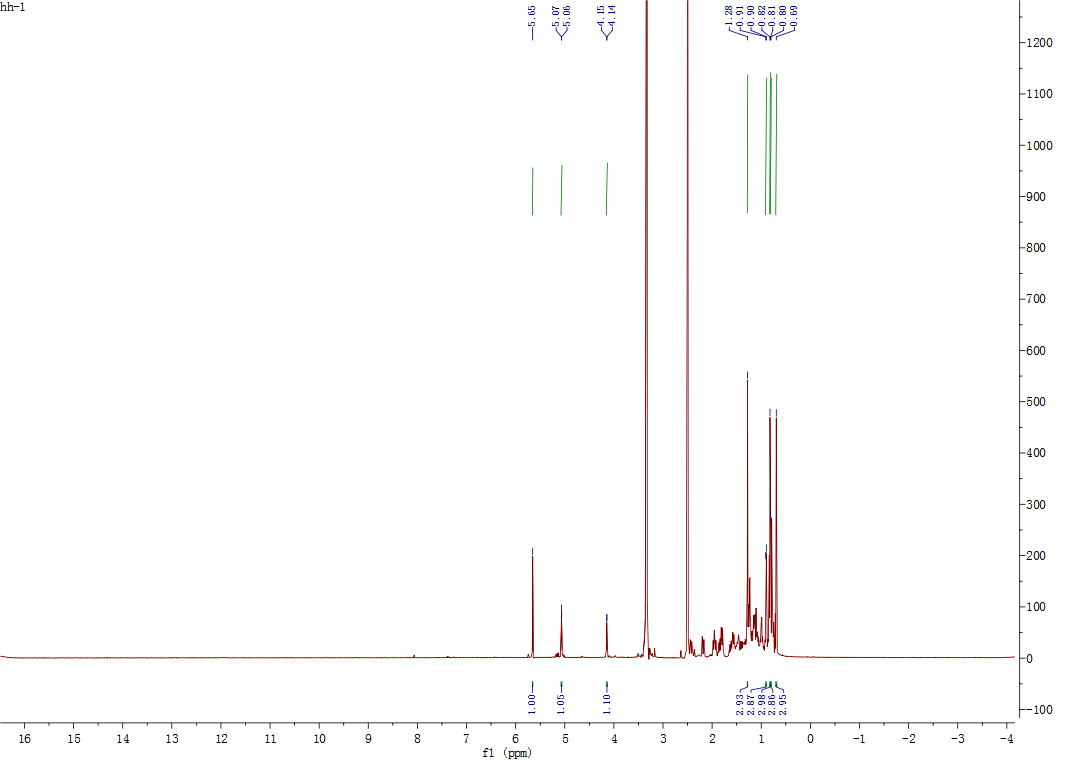

Supplement: Supplementary file 2 — Figure S2 [file FSN3-11-5166-s001.tiff]

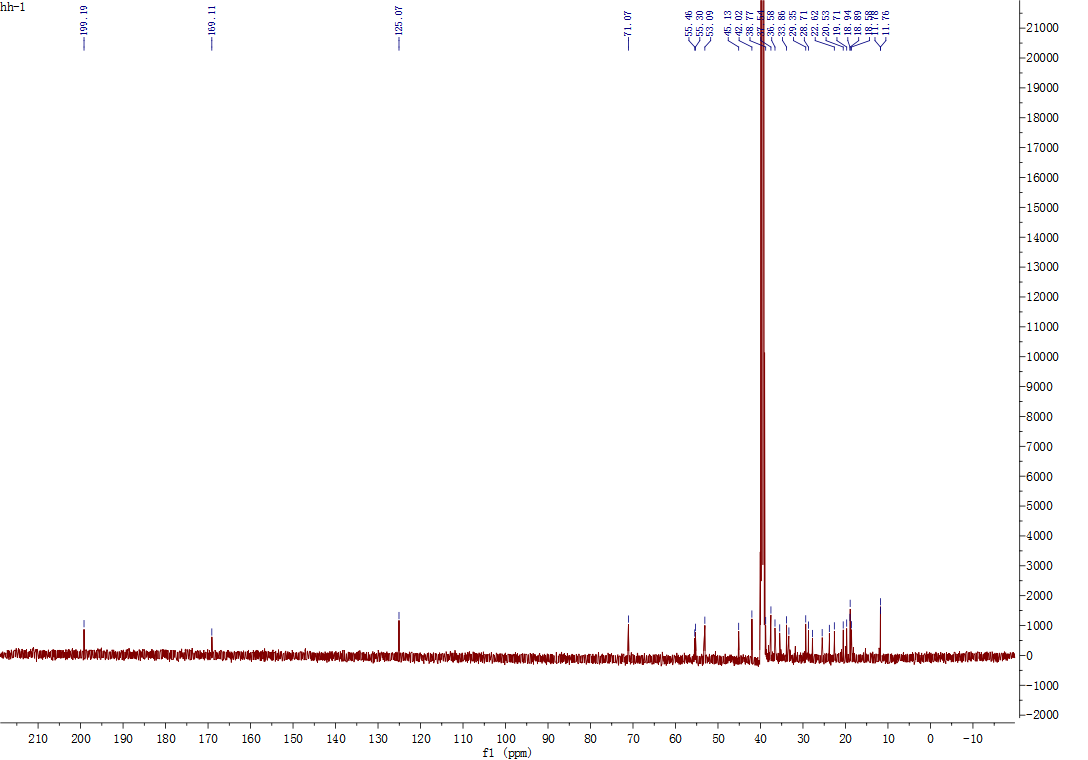

Supplement: Supplementary file 3 — Figure S3 [file FSN3-11-5166-s002.tiff]

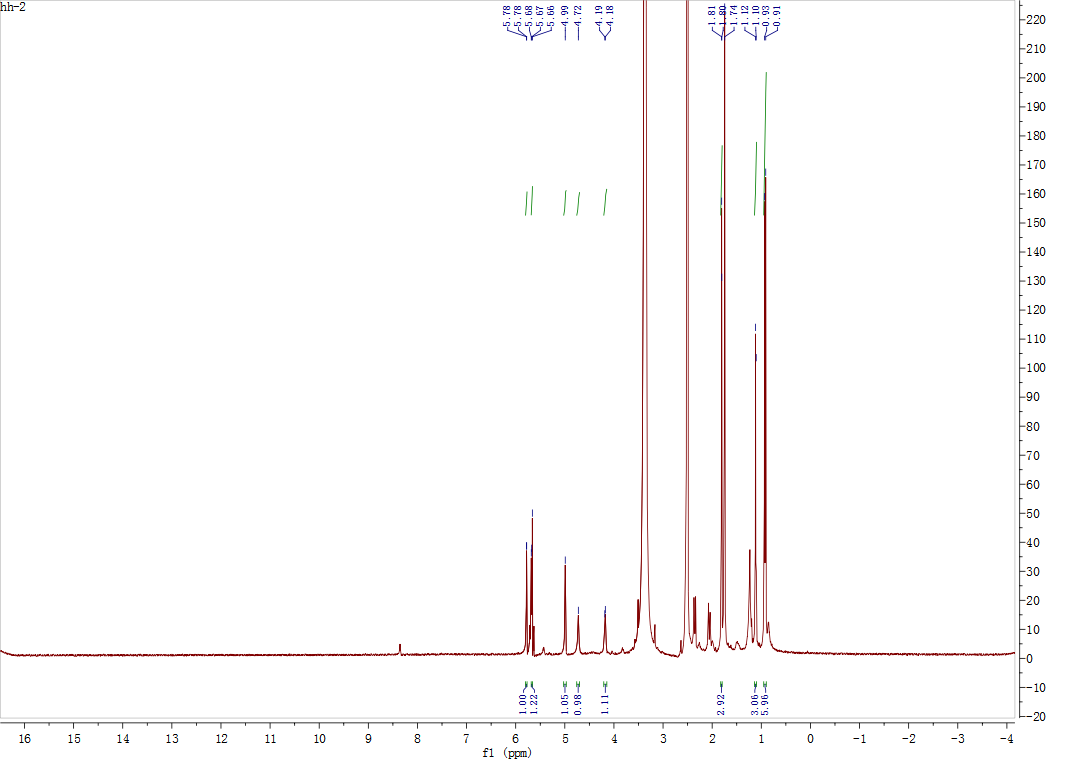

Supplement: Supplementary file 4 — Figure S4 [file FSN3-11-5166-s006.tiff]

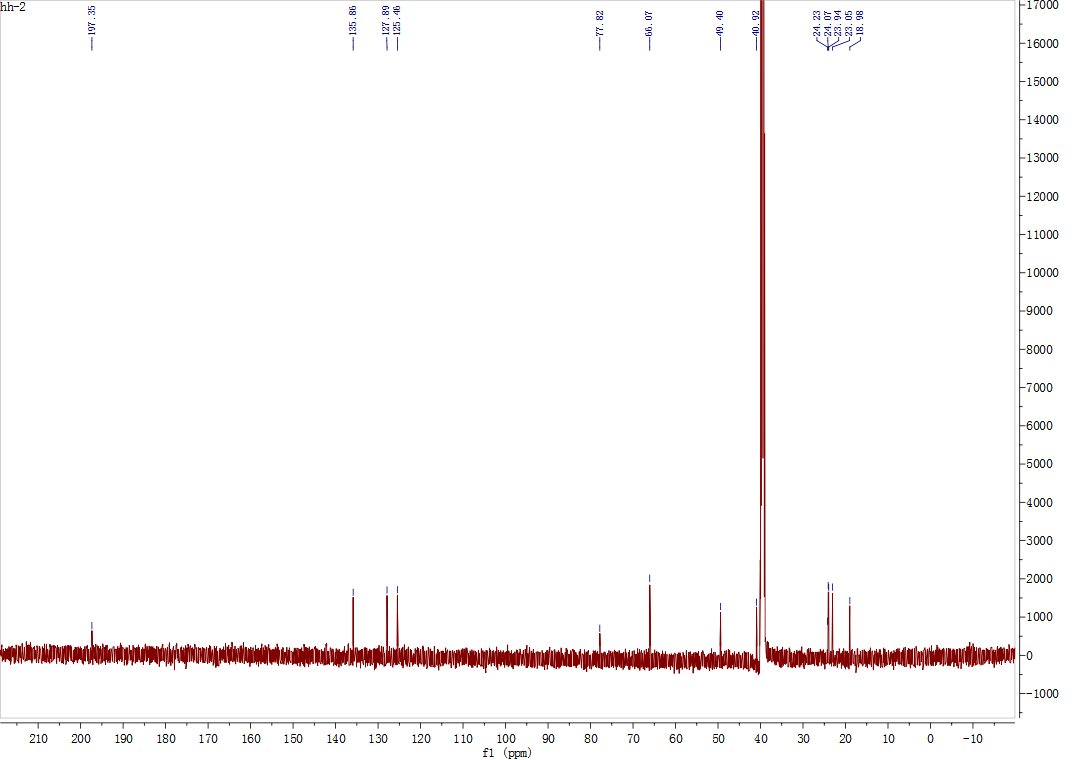

Supplement: Supplementary file 5 — Figure S5 [file FSN3-11-5166-s005.tiff]

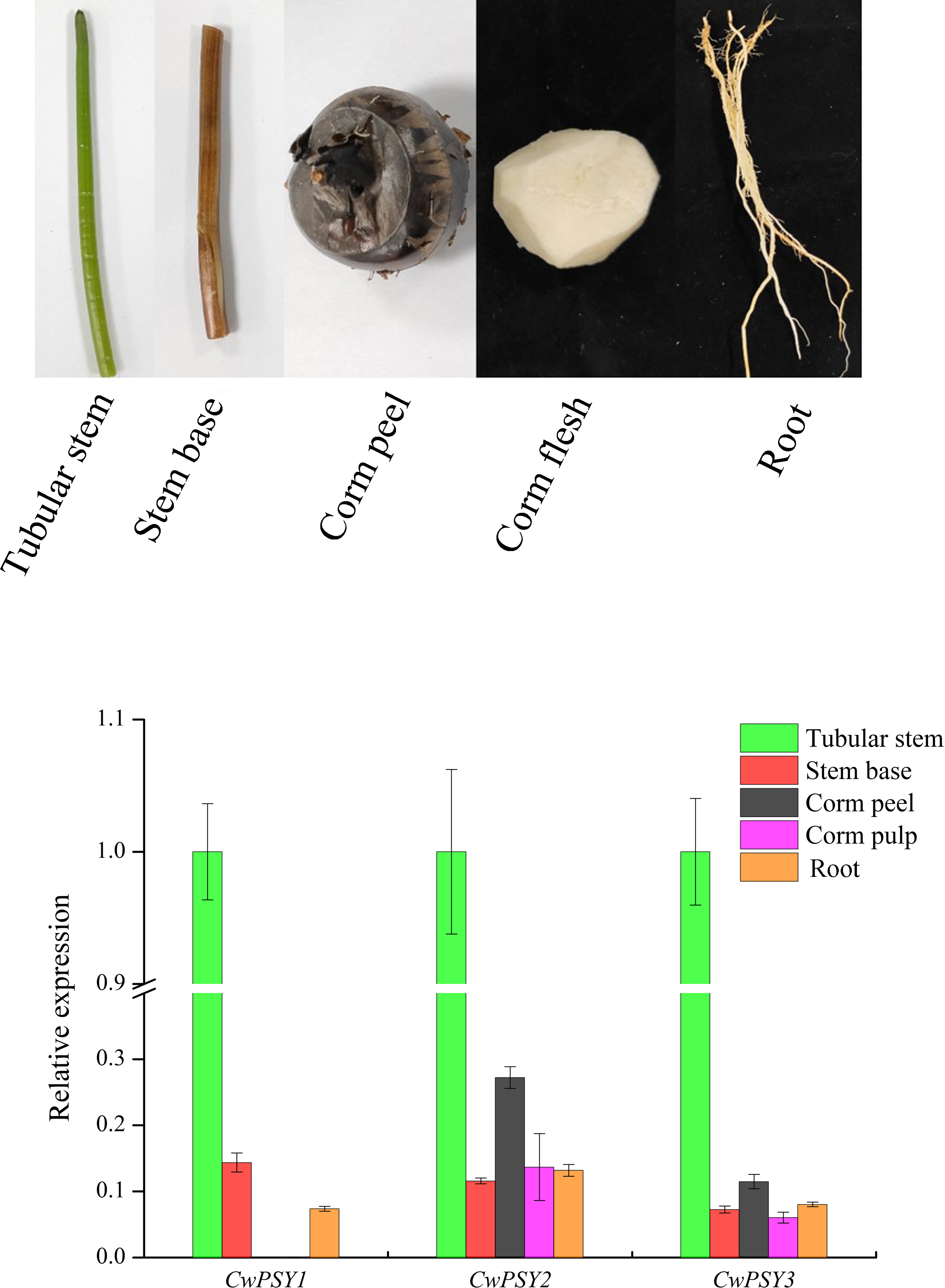

Supplement: Supplementary file 7 — Figure S7 [file FSN3-11-5166-s008.jpg]
