# Supplementary material for: Identification of two terpenoids that accumulate in Chinese water chestnut in response to fresh‐cut processing
Source: Food Sci Nutr. 2023 Jun 12;11(9):5166–73. doi: 10.1002/fsn3.3475 (PMC10494652; doi:10.1002/fsn3.3475)
Supplement: Supplementary file 6 — Figure S6 [file FSN3-11-5166-s009.pdf]

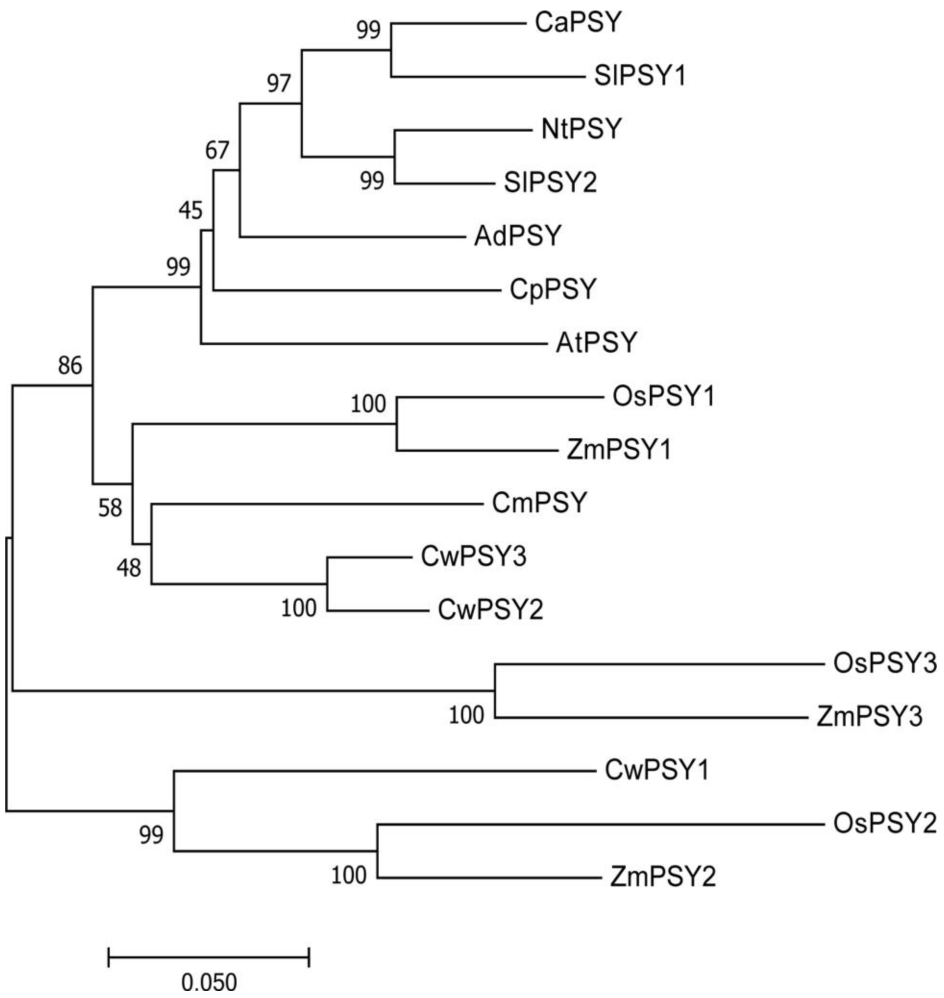

The tree was constructed using the neighbor-joining method with MEGA 7.0 software. Species and GenBank accession numbers are as follows: AdPSY, ACO53104 (*Actinidia deliciosa*); AtPSY, AAM62787 (*Arabidopsis thaliana*); CaPSY, P37272.1 (*Capsicum annuum*); CpPSY, ABG72805 (*Carica papaya*); CmPSY, AEB91323 (*Clivia miniata*); NtPSY, ADK25054 (*Nicotiana tabacum*); OsPSY1, AAS18307; OsPSY2, AAS17009; and OsPSY3, ACI62767 (each *Oryza sativa*); SlPSY1, NP_001234671; SlPSY2, NP_001234812 (both *Solanum lycopersicum*); ZmPSY1, AAR08445; ZmPSY2, NP_001108117; and ZmPSY3, NP_001108125 (each *Zea mays*).
