# Supplementary material for: Identification of two terpenoids that accumulate in Chinese water chestnut in response to fresh‐cut processing
Source: Food Sci Nutr. 2023 Jun 12;11(9):5166–73. doi: 10.1002/fsn3.3475 (PMC10494652; doi:10.1002/fsn3.3475)
Supplement: Supplementary file 8 — Figure S8 [file FSN3-11-5166-s003.pdf]

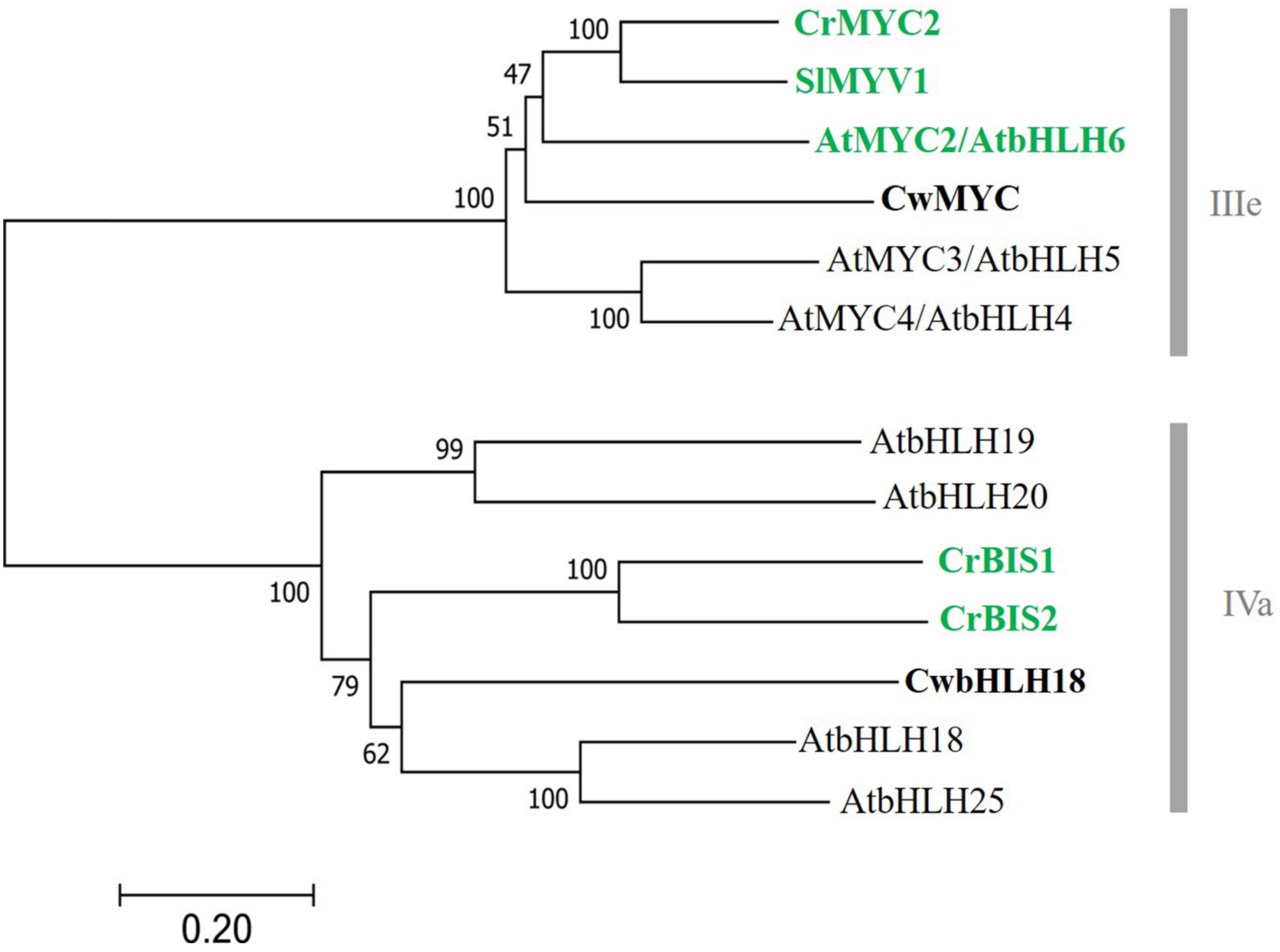

The phylogenetic tree was constructed with the neighbor-joining method. Bootstrap values were calculated as a percentage of 1000 replicates. The detailed information for bHLHs used here are as follows: CrMYC2, AF283507; SlMYC2, KF430611; CrBIS1, KM409646; CrBIS2, KM409645; The amino acid sequences of AtbHLHs are downloaded from the Arabidopsis thaliana Transcription Factor database (http://plntfdb.bio.uni-potsdam.de/v3.0/).
