# Supplementary material for: Identification of two terpenoids that accumulate in Chinese water chestnut in response to fresh‐cut processing
Source: Food Sci Nutr. 2023 Jun 12;11(9):5166–73. doi: 10.1002/fsn3.3475 (PMC10494652; doi:10.1002/fsn3.3475)
Supplement: Supplementary file 9 — Table S1 [file FSN3-11-5166-s007.pdf]

Target	Forward primer(5′-3′)	Reverse primer(5′-3′)
CwDXS	CTTGAGGTAGGTAAAGGGAGGATAC	GGGCCTCCACTTTATATTACCATC
CwDXR	GTCACCGTAGTTACAGGGATAGTAG	TAGAATTATCCTCCTGAGTGCTCC
CwCMK	CAGGAAGTGGAAGCACAATTGTAG	CTAGCACCGGAAACAAATACATCC
CwMDS	AATAGATATTCCTCATGACCGGGG	GATCAAAGTGGCATCTAGGTTACC
CwHDS	GCACTCTACTTATGGATGGATTGG	CTCCTCTGTAATCTACTTCTTCCCC
CwHDR	GGACAATGATGCAAAGGTATGGAG	CCATCCTCCAACTACTAGTATCAGG
CwHMGS	GGCAACATCCTTTCAGTCTCTCTA	CTTGTCAGATAGTAGGTACCAGGAG
CwHMGR	CTATGAATCAATCCTCGGACAGTG	CCATCTCTCAGAACCACACTAGTAG
CwPMK	CCTCAAGTACTCTCTTCTACTCAGG	GAGTAGATGAACCTCCAGTACCAG
CwMVD	GGGCCATGGGAAATAATACTGATG	CTAGTCTCCTTCTGCTTTGAACTC
CwPSY1	TTGTACTGCTATTACGTGGCTGGTA	CAGCAAATATGTCATCCTCACTCAG
CwPSY2	ATCACCAAGGGTATGTTCACCGGTTG	GGTCCGAAATCGAGATCCGTACTACTC
CwPSY3	GTTGCCACGGCTCTGTTCACCAGTC	CACACAAGCATACTTCCAGTTGGTATG
CwPDS	CACATGCAAGGAGTATTATGACCC	CTCTGGTCAGCAGATATCTCATCAG
CwZDS	CTAAGGTGGGGTTGTAGACAGATA	TGATCTCCCTACTTGTAGCCTTAG
CwMYC	GGTGCAATGTAATAAAAAGAACCAC	CTGAATGAAGTTGCTCTTGACTGTA
CwbHLH18	TCATAATAAAGAAATCCCAGTTCCA	CAATATCTTCACCAAAGCTCCTTTA
